# Optimal Provider Position for Video-Assisted Laryngoscopy of a Supine Patient on the Floor

**DOI:** 10.7759/cureus.82505

**Published:** 2025-04-18

**Authors:** Jeffrey S Lubin, Justin Brooke, Mohit S Bhide

**Affiliations:** 1 Department of Public Health Sciences, Penn State College of Medicine, Hershey, USA; 2 Department of Emergency Medicine, Penn State Health Milton S. Hershey Medical Center, Hershey, USA; 3 Department of Emergency Medicine, Denver Health and Hospitals, Denver, USA; 4 Department of Internal Medicine, University of Pittsburgh Medical Center, Pittsburgh, USA

**Keywords:** intubation simulation, manikin, rescuer position, video laryngoscopy (vl), endotracheal intubation

## Abstract

Introduction

This study presents a direct comparison of prone, kneeling, and straddling provider positions during endotracheal intubation of a supine patient on the ground, focusing on success rates, time, and ease of intubation.

Methods

Forty-five prehospital providers from a single Emergency Medical Services (EMS) program performed intubations on a manikin using a McGrath video laryngoscope (Medtronic, Minneapolis, USA) in prone, kneeling, and straddling positions. Each provider had three attempts per position, with success defined as the endotracheal tube passing through the vocal cords. Wilcoxon signed-rank tests and Bonferroni corrections were used for statistical analysis.

Results

The kneeling position had the highest success rate (97.8% on all three attempts), followed by the prone position (91.1%). The straddling position had the lowest success rate (66.7%). Median intubation times were 8.8 seconds for prone, 9.8 seconds for kneeling, and 22.0 seconds for straddling. Statistically significant differences were found between the straddling position and both the kneeling and prone positions (p < 0.001).

Conclusion

Providers were most successful and efficient in the kneeling and prone positions for intubating patients on the ground. The straddling position was the least effective and required more time. These findings suggest that training programs should emphasize kneeling and prone positions to improve prehospital airway management.

## Introduction

Emergency airway interventions are crucial for the resuscitation of acutely ill and injured patients. Endotracheal intubation (ETI) is recognized as the gold standard for airway management and has been performed an estimated 265,000 times annually in the United States over the last 25 years. Despite its importance, the procedure is fraught with challenges, especially in the prehospital setting [1,2]. ETI is often associated with adverse events such as tube misplacement, multiple intubation attempts, and iatrogenic hypoxia [3-6]. Furthermore, emergency ETI procedures can delay other aspects of resuscitation, including lengthy interruptions in chest compressions during cardiopulmonary resuscitation [7]. Success rates and time to intubation are critical performance metrics for evaluating technique, particularly in challenging scenarios.

These challenges are compounded by the suboptimal conditions in which prehospital providers operate, characterized by significant scene distractions and limited space. Patients are most often supine [8]. Although ETI is traditionally performed with the patient at the provider’s xiphoid process, prehospital patients frequently require modified intubation approaches to accommodate unusual positioning. These approaches include emergency personnel assuming prone, sitting, kneeling, straddling, and left lateral decubitus positions relative to the patient. Previous studies have indicated that all tested positions may be satisfactory for intubating a patient on the ground, with the straddling position requiring more time than the kneeling position and the left lateral decubitus position requiring fewer ETI attempts [9,10]. Despite these findings, there is limited research directly comparing the success rates and efficiency of different provider positions during ETI in these settings [11-17].

This study aims to address this gap by directly comparing the success rates and time efficiency of ETI performed by prehospital providers in kneeling, prone, and straddling positions. We hypothesize that the kneeling position will yield the highest success rates and shortest intubation times, thereby reducing the risk of hypoxemia, esophageal intubations, and pulmonary aspiration.

## Materials and methods

The study was reviewed and approved by our university's Institutional Review Board (Penn State College of Medicine Institutional Review Board approval no. STUDY00008281).

A convenience sample of paramedics and prehospital registered nurses from a single Emergency Medical Services program was used for the study. After obtaining demographic information regarding years of experience and an estimated number of career intubations, participants were asked to intubate a manikin placed supine on the floor using a McGrath video laryngoscope (Medtronic, Minneapolis, USA) while in one of three different body positions: prone, kneeling, and straddling. No additional training was given to the providers on the use of the McGrath video laryngoscope prior to their participation in the study.

All intubations were performed in the same room with the same lighting and surface conditions. In the prone position, participants lay on their stomachs with elbows raised behind the head of the manikin. In the kneeling position, participants knelt behind the head of the manikin. For the straddling position, participants placed their knees between the manikin's arms and torso, holding the laryngoscope in the right hand. Each participant was given three attempts to intubate in each of the positions. The time to intubation was recorded by a trained observer using a stopwatch, starting when the participant picked up the laryngoscope and stopping when the participant indicated correct placement of the endotracheal tube. Successful intubation was defined as the endotracheal tube passing through the manikin’s vocal cords, confirmed by lung inflation with ventilation.

Data regarding the time until successful intubation was recorded, with a 60-second time limit for each attempt. Attempts lasting more than 60 seconds were considered unsuccessful. The number of successes out of three attempts was tabulated, and these variables were the outcomes used for comparisons. All data were entered into a Microsoft Excel spreadsheet (Microsoft Corporation, Redmond, USA) for analysis.

Due to the paired and skewed nature of the data, Wilcoxon signed-rank tests were used to compare original and recoded time variables between intubation position groups. To control for multiple comparisons, p-values were adjusted using the Bonferroni correction. The average, first attempt, and third attempt times were analyzed to ensure significance was not found by chance. To compare success counts within each group, a generalized estimating equations (GEE) model was utilized to analyze binary, ordinal, or count outcome variables with repeated measures for the same subject. Poisson regression was employed to allow for count outcomes. The GEE Poisson model provided frequencies of success counts within each group, overall p-values, and inter-group odds ratios.

## Results

A total of 45 prehospital providers participated in our study, including nine registered nurses and 36 paramedics. They averaged 20 years of experience, with 7.6 years at the EMS agency, and performed an estimated 221 intubations over their careers. On average, each provider estimated they had intubated seven times in the past year.

Table 1 summarizes the participants’ preferred intubating positions for a patient supine on the floor. Prone was the preferred position, while straddling was the least preferred.

**Table 1:** Provider preference for intubation position

Preferred Intubation Position	Frequency % (n) (n=45)
Prone	64.4% (29)
Kneeling	31.1% (14)
Straddling	4.4% (2)

Table 2 shows the frequency of successful intubations out of three attempts in the three different positions. The kneeling group had the highest success rate, with 97.8% achieving success on all three attempts. The prone group followed with a 91.1% success rate on all three attempts. The straddling group had the lowest success rate, with only 66.7% achieving success on all three attempts

**Table 2:** Frequency of successful intubations in varying positions by percent and total number

	Successful Intubations % (n) (n=135)			
Attempts	0/3	1/3	2/3	3/3
Kneeling	2.2% (1)	0% (0)	0% (0)	97.8% (44)
Prone	2.2% (1)	0% (0)	6.7% (3)	91.1% (41)
Straddling	4.4% (2)	6.7% (3)	22.2% (10)	66.7% (30)

**Figure 1:**
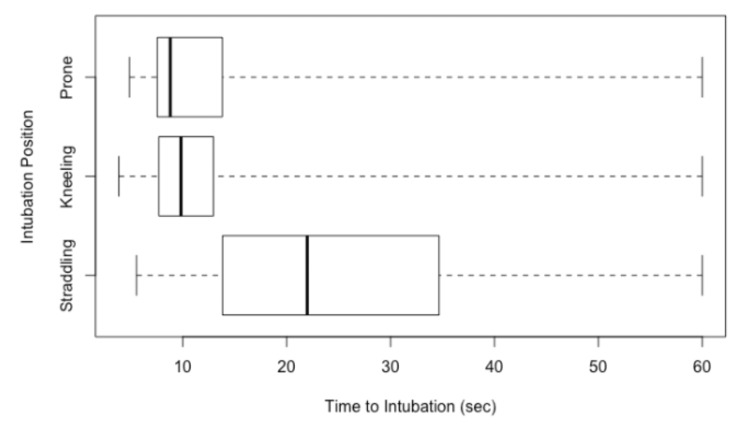
Comparison of time to intubation between the three rescuer positions

As shown in Figure 1, the median time to successful intubation was highest in the straddling group (22.0 s, IQR: 20.8-34.7), compared to the kneeling (9.8 s, IQR: 5.3-13.0) and prone groups (8.8 s, IQR: 6.3-13.8). Participants intubated faster while kneeling compared to straddling (p < 0.001) and also faster while prone versus straddling (p < 0.001). There was no statistically significant difference in intubation time between the kneeling and prone positions (p = 1.0).

## Discussion

Prehospital care often occurs in unconventional settings, requiring airway management in diverse environments such as hospital beds, cars, ambulances, or living rooms. Given the potential for non-standard patient positioning, multiple approaches, such as kneeling, straddling, and prone, can be used to perform intubation successfully.

Previous studies have investigated optimal provider positions using a direct laryngoscope. Our study, however, used the McGrath video laryngoscope to evaluate kneeling, prone, and straddling positions and their impact on ETI success and time to successful intubation for a patient supine on the floor. We found no statistical difference between the kneeling and prone positions in terms of successful intubations, with the kneeling position performing the best and the prone position following closely. The straddling position statistically performed the worst when compared to the prone and kneeling positions. These findings highlight the advantages of performing ETI from a kneeling or prone position over a straddling position.

Furthermore, time to successful intubation was statistically equivalent for the kneeling and prone positions, with the prone position performing slightly better. The straddling position again performed the worst statistically compared to the prone and kneeling positions. This finding is remarkable, as it demonstrates that it takes half as much time, on average, to successfully intubate a patient from a kneeling or prone position rather than from a straddling position. However, the straddling position has several significant advantages. It can be optimal for situations where the patient is inaccessible for standard intubation, such as being trapped in a car or during air medical transportation [15,16]. Additionally, the technique is performed with the laryngoscope in the right hand and utilizes shoulder extension and arm flexion, producing a larger force to manipulate the soft tissues.

Notably, kneeling and prone positions offer the highest intubation success rates with the least amount of time to success relative to the straddling position, suggesting that performance is affected by the rescuer's position. There were no statistical differences between kneeling and prone positions with respect to performance or time to successful intubation, suggesting that either approach offers benefits. These results are congruent with previous evidence, which found statistical differences between straddling and prone positions, with straddling outperforming in time to successful intubation. The differences in intubation success rates and times between positions can be attributed to physiological and mechanical factors. Kneeling and prone positions may allow for better visualization of the glottis and more practical application of force, leading to higher success rates. Understanding these mechanisms can inform training and practice, ensuring providers have the knowledge to choose the most effective position in any given scenario. Despite evidence suggesting that the straddle position is inferior to kneeling or prone positions, it is advantageous in cases where visualization of the glottis is difficult, as the straddle position confers more reserve force than the prone position and can lead to quicker intubation with fewer attempts [17].

The findings of this study have significant implications for prehospital care and emergency medical services. Training programs should incorporate these techniques, ensuring providers are well-prepared to manage airways in unconventional settings, and may need to emphasize the advantages of kneeling and prone positions. Additionally, equipment design could be optimized to support these positions, enhancing the efficiency and effectiveness of airway management in diverse settings. According to a recent guideline, the use of video laryngoscopes, such as the McGrath, has been recommended to improve intubation success rates in prehospital settings.

While our study aligns with previous research in some aspects, it also presents unique insights. For instance, using the McGrath video laryngoscope may offer advantages over traditional direct laryngoscopes, suggesting a need for further comparative studies. Conflicting results in the literature highlight the importance of considering various factors, such as provider experience and patient condition when evaluating intubation techniques. A systematic review and meta-analysis found that physician providers had higher intubation success rates than non-physician providers, emphasizing the need for specialized training [18].

Beyond success rates and intubation times, the impact of different positions on patient outcomes should be considered. Factors such as patient comfort, potential for injury, and long-term recovery are crucial in evaluating the overall effectiveness of intubation techniques. Future studies should explore these aspects to provide a comprehensive understanding of the best practices for airway management.

While our study provides valuable insights, it has limitations. The sample size and specific study conditions may affect the generalizability of the findings. Also, the lack of randomization in the attempt sequence (e.g., prone-kneeling-straddling) could have resulted in learning effects, potentially influencing performance. As this was a manikin study, it could not evaluate factors like glottic view grade, force required, or ergonomic strain, which may also affect real-world performance. Furthermore, while we suspect that the kneeling/prone positions may offer a better glottic view due to mechanical advantage, these forces were not directly measured. Future research should aim to replicate these results in larger, more diverse populations and explore additional variables such as provider fatigue and patient anatomy. Additionally, the development of evidence-based guidelines for prehospital airway management can help standardize practices and improve outcomes.

## Conclusions

The findings of this study suggest that kneeling and prone positions are superior to the straddling position for endotracheal intubation of supine patients on the ground. These results have significant implications for prehospital care and emergency medical services, suggesting that training programs should emphasize these positions. However, it should be acknowledged that the findings are simulation-based and require real-world validation. Future research should replicate this study with a larger, more diverse sample and explore the impact of different positions on patient outcomes, including comfort and recovery. Developing evidence-based guidelines for prehospital airway management can help standardize practices and improve overall patient care.
